# Proximity to public green spaces and depressive symptoms among South African residents: a population-based study

**DOI:** 10.1186/s12889-024-18385-1

**Published:** 2024-03-29

**Authors:** Busisiwe Shezi, Hilbert Mendoza, Darshini Govindasamy, Lidia Casas, Yusentha Balakrishna, Jason Bantjes, Renée Street

**Affiliations:** 1 Environment and Health Research Unit, South African Medical Research Council, 491 Peter Mokaba Ridge, Morningside 4091 Durban, South Africa; 2 Department of Environmental Health, Faculty of Health Sciences, University of Johannesburg, Corner Siemert and Beit Street, Doornfontein 2028 Johannesburg, South Africa; 3 Social Epidemiology and Health Policy, Department of Family Medicine and Population Health, University of Antwerp, Campus Drie Eiken, Doornstraat 331, BE-2610 Wilrijk, Belgium; 4 Health Systems Research Unit, South African Medical Research Council, Francie van Zijl Drive, Parow Valley 7501 Cape Town, South Africa; 5 Biostatistics Research Unit, South African Medical Research Council, 491 Peter Mokaba Ridge, Morningside 4091 Durban, South Africa; 6 Mental Health, Alcohol, Substance Use and Tobacco Research Unit, South African Medical Research Council, Francie van Zijl Drive, Parow Valley, Cape Town, South Africa 7501; 7 Department of Psychiatry and Mental Health, University of Cape town, Groote Schuur Drive, Observatory 7925 Cape Town, South Africa; 8 Environment and Health Research Unit, South African Medical Research Council, Francie van Zijl Drive, Parow Valley 7501 Cape Town, South Africa

**Keywords:** Depression, Green spaces, Proximity to public green spaces, Low-and middle-income countries, South Africa

## Abstract

**Background:**

Exposure to green spaces has been suggested to improve mental health and may reduce the risk of depression. However, there is generally limited evidence on the association between green spaces and depression originating from low-and middle-income countries and Africa in particular. Here, we investigate the association between proximity to public green spaces and depressive symptoms among residents of Gauteng Province, South Africa.

**Methods:**

We used data from the 2017/2018 Gauteng quality of life survey. We included all individuals aged 18 years or older residing in the nine municipalities of Gauteng Province that completed the survey (*n* = 24,341). Depressive symptoms were assessed using the Patient Health Questionnaire-2. Proximity to public green spaces was defined as self-reported walking time (either less or greater than 15 min) from individuals’ homes to the nearest public green space. To assess the association between access to public green spaces and depressive symptoms, we used mixed-effects models, adjusted for age, sex, population group (African, Indian/Asian, Coloured (mixed race), and White), educational attainment, and municipality. We additionally performed stratified analyses by age, sex, educational attainment, and population group to evaluate whether associations differed within subgroups. Associations are expressed as prevalence ratios (PR) and their 95% confidence intervals (95% CI).

**Results:**

We observed a 6% (PR = 0.94, 95%CI = 0.92–0.96) prevalence reduction in depressive symptoms for individuals who reported that the nearest public green space was less than 15 min from their homes as compared to those who reported > 15 min. After stratification, this inverse association was stronger among females, individuals aged 35–59 years,those with higher levels of educational attainment, and Coloured individuals as compared to their counterparts.

**Conclusion:**

Our findings suggest that public green spaces close to residential homes may be associated with a reduction in the occurrence of depressive symptoms among urban populations in resource-constrained settings like South Africa.

**Supplementary Information:**

The online version contains supplementary material available at 10.1186/s12889-024-18385-1.

## Introduction

Depression is a global public health threat. The World Health Organization (WHO) estimates that in 2019, approximately 280 million individuals were living with depressive disorders [1]. In addition, the majority of these cases originated from low- and middle-income countries such as South Africa [1]. In South Africa, the estimated national prevalence of depression ranged from 14.7 to 38.8% in 2021 [2]. As a consequence of this high prevalence, depressive illnesses are associated with negative outcomes such as a high economic burden to manage depression and increased risk of occurrence of comorbid health outcomes such as cardio-vascular diseases, and cancer among others [3].

Epidemiological studies have identified individual-level risk factors associated with depression, including genetics, adverse childhood experiences, health lifestyle behaviors (smoking and alcohol drinking), and female gender [4, 5]. However, an increasing number of studies have also begun to examine the link between environment-level factors such as the natural environment (green spaces like parks and forests) and depression [6–8].

Diverse mechanisms have been suggested through which green spaces may influence the onset and course of depression. Firstly, increased access and availability of green spaces promotes social cohesion [9], outdoor physical activity [10, 11], and reduction of stress [12, 13], all of which reduce the risk of depression. Secondly, green spaces aid in the reduction of environmental stressors such as air pollutants [14, 15], and noise [16, 17], both of which have been shown to increase the risk of depression [18, 19].

To date, the epidemiological evidence regarding the relationship between green spaces and depression has majorly originated from high-income countries [7, 20]. However, it cannot be assumed that findings from high-income countries are generalizable to African low- and middle-income countries like South Africa owing to the differences in urban conditions, cultural, and environmental factors that exist between the two settings and given that the aetiology of depression differs across economic regions [21]. To our knowledge, there have been only two similar studies that have been conducted in African low-and middle-income countries [22, 23]. Zewdie et al., (2022) observed no statistically significant association between green spaces and depressive symptomology among young adults in Ethiopia. Conversely, Tomita et al. (2017) observed statistically significant inverse associations between green spaces and depressive symptoms among middle-income individuals and those who resided in rural areas of South Africa. Owing to inconsistencies in the available evidence, there is thus a need for more studies to be conducted. Importantly, the two aforementioned studies used normalized difference vegetation index (NDVI); a quantitative measure of the available amount of surrounding greenness. However, NDVI is unable to capture other important qualitative characteristics related to green spaces such as proximity [24]. Proximity (walking time) to public green spaces has been shown to influence the utilisation of the available green spaces [25, 26] which in turn may also affect the risk of depression. Consequently, there is a need to investigate how proximity to green spaces influences depression occurrence among individuals of African low-and middle-income countries.

South Africa is one of the most urbanized countries in Africa with around 67% of its population living in urban areas and is projected to increase to 80% by 2050 [27]. There is thus a potential for natural environments such as green spaces to drastically diminish. For example, a study conducted in the African region reported a reduction in green space of 51.8%, with an annual loss rate of 3.9% between 2003 and 2016. Meanwhile, the built-up area increased by 562.1%, experiencing a 43.2% annual growth rate. Specifically, the study showed that approximately 1410.7 hectares of green space were converted to built-up areas during the latter period [28].

Consequently, it is important to understand the potential effects of such environments on depression. Here, we present a cross-sectional study where we investigate the association between proximity to public green spaces and depressive symptoms among South African residents of Gauteng Province. In addition, we assess whether the aforementioned association differs by socio-demographic characteristics (sex, age, educational attainment, and population group).

## Methods

### Study design, and population

This study utilized a cross-sectional study design based on population-level data from the Gauteng quality of life survey. The quality of life survey is conducted by the Gauteng City Region Observatory (GCRO) in partnership with government and research institutions. The GCRO was established in 2008 as a partnership between the University of Johannesburg, the University of the Witwatersrand, and the Gauteng local and provincial government [29]. The biennial quality of life survey was first conducted in 2009, and it measures the quality of life, socio-economic circumstances, attitudes to service delivery, psycho-social-attitudes, and other characteristics in the Gauteng province [30]. The current study draws from the 2017/18 quality of life survey. The selection of participants and sampling strategies are described elsewhere [31]. In brief, participants aged 18 years or older residing in one of the nine municipalities of Gauteng Province were eligible for enrollment into the survey which took place between 31st October 2017 and 7th September 2018. Figure S1 of the supplementary material is a map that illustrates the positioning of the aforementioned municipalities within Gauteng and the positioning of Gauteng within the African continent. A detailed map showing the relative positions of where these participants resided in the nine municipalities is illustrated elsewhere [32]. For this study, we excluded individuals who had “other” as their population group (*n* = 85, 0.34%) because this sub-group was too small to analyze meaningfully, and those with missing covariate information (*n* = 463,1.87%).

### Depressive symptoms

Depressive symptoms in the past two weeks prior to the start of the survey were assessed using the Patient Health Questionnaire-2 (PHQ-2). The PHQ-2 is a self-report screening instrument that consists of the first 2 items (anhedonia and depressed mood) of the original Patient Health Questionnaire-9. For each of the 2 items, participants choose between four possible response options ranging on a Likert scale from 0 (not at all), 1(a few days), 2 (more than half the days), and 3 (nearly every day). Total scores for the PHQ-2 range from 0 to 6, with higher scores indicating more severe symptoms of depression. Following a previous study, we transformed the PHQ-2 scores into a binary variable (depressive symptoms vs. no depressive symptoms) applying a threshold value of ≥ 2, reflecting being at a higher risk for depression with a sensitivity and specificity of 0.91 and 0.67 respectively [33].

### Proximity to public green spaces

Proximity to public green spaces was defined as the self-reported walking time from individuals’ homes to the nearest park or green space. The 2017/18 quality of life survey questionnaire [30] included a close-ended yes/no question (“Which of the following can you walk to within 15 minutes of this dwelling? Park or green public space”) which we used to assess proximity to parks. We dichotomized proximity to public green spaces to within 15 min and greater (>) than 15 min for those who reported “yes” and “no” respectively.

### Potential confounders

Potential confounders were identified a priori based on previous similar studies [34]. Figure S2 of the supplementary material is a directed acyclic graph that illustrates the relationship between our included confounders in the association between proximity to public green spaces and depressive symptoms. These included: sex (male and female), population group (African, Indian/Asian, Coloured (Coloured refers to people of mixed race), and White), age, and individual socio-economic status. Age was categorized according to thnational statistics service of South Africa census guidelines [35] into three categories namely; youth (18–34 years), adults (35–59 years), and the elderly (≥ 60 years). Individual socio-economic status was approximated by the highest level of educational attainment namely; no education, primary education (grade 0-grade 7), secondary education (grade 8-grade 12), and tertiary education (college certificate, college diploma, university degree, and post-graduate degree). With regards to population group, the apartheid regime implemented a hierarchical racial classification system, categorizing people based on race. In recognition of the consequences of colonialism and apartheid, which includes ongoing inequalities in access to education, employment, and healthcare, we grouped the population into the following categories: African, Indian/Asian, Coloured, and White [36, 37]. These categories are also used in official government policies which are also used for census purposes in South Africa.

### Statistical analyses

We used mixed-effects models to investigate the relationship between proximity to public green spaces and depressive symptoms. We included a random effect variable (municipality) in our models to account for the unobserved between-municipality heterogeneity. We built our main model using a stepwise approach, which involved progressively adjusting for confounders in successive models. Model one (M1) accounted for between-area variability by including a random term (municipality). Model two (M2) was M1 adjusted for confounders (sex, age, and population group). Model three (M3) was our main model and it was M2 further adjusted for educational attainment. We present our effect estimates as prevalence ratios (PRs) with their 95% confidence intervals (95% CIs).

We conducted stratified analyses by four socio-demographic characteristics (sex, age groups, educational attainment, and population group) to evaluate whether associations between proximity to public green spaces and depressive symptoms differed within the subgroups of these socio-demographic characteristics.

Finally, we performed several sensitivity analyses to assess the robustness of our findings. Firstly, we applied a PHQ-2 threshold score value of ≥ 3 to examine the potential misclassification of depressive symptoms. Previous similar studies used this threshold value for the classification of depressive symptoms [38, 39]. Secondly, to account for those with missing covariate data, we performed multiple imputation by chained equations to generate five complete datasets, and our main model was rerun on the full imputed population. Thirdly, we adjusted for seasonal differences in data collection as a potential confounder in the association between proximity to public green spaces and depressive symptoms. We did this by transforming the month of data collection for each individual into one of the four seasons experienced in South Africa (spring = September to November, summer = December to February, autumn = March to May, and winter = June to August). Fourthly, we adjusted for age in its continuous form rather than the categorical form we used in our main model. Lastly, we used continuous PHQ-2 scores as adopted in a previous study [40] and used negative binomial regression models to assess the association between proximity to public green spaces and depressive symptoms. All the aforementioned statistical analyses were done using Stata version 17 (StataCorp, College Station, TX, USA).

## Results

A total of 24,341 individuals were included in our analyses. Table 1 presents the distribution of proximity to public green spaces and socio-demographic characteristics for the total population and depressive symptoms. In total, 9,099 individuals had a total score of ≥ 2 in the PHQ-2, suggesting they were at risk of depression; presenting a prevalence proportion of 38 cases per 100 individuals within our study population. The prevalence of depressive symptoms was higher in females (58.7%) as compared to males (41.3%). Approximately half of the total population (52.2%) reported that they had to walk more than 15 min from their homes to the nearest public green space. With regards to the socio-demographic characteristics, the mean age was 40.7 years (standard deviation(SD) = ± 14.9); by depressive symptoms, the mean age for those who reported depressive symptoms (42.5 ± 15.8) was higher than those who did not report depressive symptoms (39.5 ± 14.2). The majority of the total population (60.9%) had completed secondary education.

**Fig. 1:**
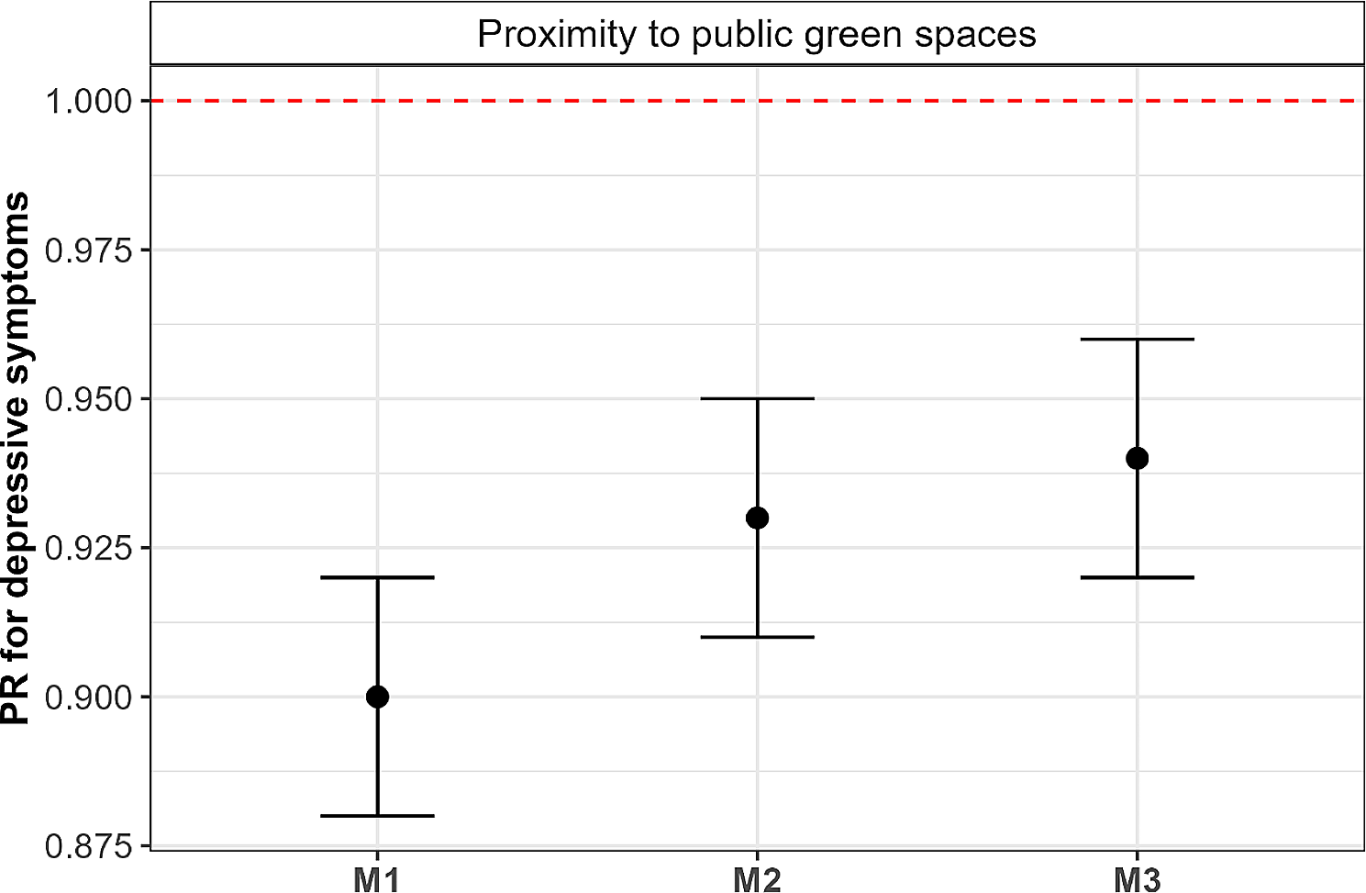
Prevalence Ratios (PR) and 95% confidence intervals (CI) for those who reported within a walking time of 15 min to the nearest public green space [reference group: >15 min] with increasing degree of adjustment of confounders for the association between proximity to public green spaces and depressive symptoms. Note **M1**: Accounted for between-area variability by including a random term; **M2**: Adjusted for M1, sex, age groups, and population group; **M3**: Adjusted for M2 and educational attainment

Figure 1 illustrates the PRs and their 95% CIs for the association between proximity to public green spaces and depressive symptoms with stepwise cumulative adjustment for potential confounders for those who reported a walking time of 15 min to the nearest public green space. The exact values of the PRs and 95% CIs are presented in Supplementary Table S1. Overall, we observed an inverse association between proximity to public green spaces and depressive symptoms, however, the strength of this association slightly attenuated after adjustment for confounders (M2 and M3) but the association remained statistically significant. Results from our main model (M3) showed a prevalence reduction in depressive symptoms by 6% (PR = 0.94, 95%CI = 0.92–0.96) for individuals who reported that the nearest public green spaces were within 15 min from their homes as compared to those reported > 15 min.

**Table 1:** Proximity to public green spaces, and socio-demographic characteristics for the total population and stratified by depressive symptoms

Variable	Category	Total (*N* = 24,341)	Depressive symptoms	
			Yes (*n* = 9,099)	No (*n* = 15,242)
Proximity to public green spaces	Within 15 min > 15 min	11,625 (47.8) 12,716 (52.2)	4,051 (44.5) 5,048 (55.5)	7,574 (49.7) 7,668 (50.3)
Sex	Male Female	11,350 (46.6) 12,991 (53.4)	3,761 (41.3) 5,338 (58.7)	7,589 (49.8) 7,653 (50.2)
Age groups	18–34 years 35–59 years ≥ 60 years Mean (SD)	9,752 (40.1) 11,403 (46.9) 3,186 (13.1) 40.7 (14.9)	3,346 (36.8) 4,228 (46.5) 1,525 (16.8) 42.5 (15.8)	6,406 (42.0) 7,175 (47.1) 1,661 (10.9) 39.5 (14.2)
Educational attainment	No education Primary education Secondary education Tertiary education	683 (2.8) 2,372 (12.6) 14,828 (60.9) 6,458 (26.5)	330 (3.6) 1,094 (12.0) 5,669 (62.3) 2,006 (22.1)	353 (2.3) 1,278 (8.4) 9,159 (60.1) 4,452 (29.2)
Population group	African Coloured Indian/Asian White	20,618 (84.2) 866 (3.6) 347 (1.4) 2,510 (10.3)	7,943 (87.3) 294 (3.2) 115 (1.3) 747 (8.2)	12,675 (83.2) 572 (3.8) 232 (1.5) 1,763 (11.6)
Municipality	Ekurhuleni Johannesburg Tshwane Emfuleni Lesedi Midvaal Merafong Mogale City Rand West	6,200 (25.5) 7,688 (31.6) 4,203 (17.3) 1,690 (6.9) 454 (1.9) 514 (2.1) 977 (4.0) 1,358 (5.6) 1,257 (5.2)	1,982 (21.8) 2,821 (31.0) 1,623 (17.8) 947 (10.4) 158 (1.7) 169 (1.9) 353 (3.9) 489 (5.4) 557 (6.1)	4,218 (27.7) 4,867 (31.9) 2,580 (16.9) 743 (4.9) 296 (1.9) 345 (2.3) 624 (4.1) 869 (5.7) 700 (4.6)

**Fig. 2:**
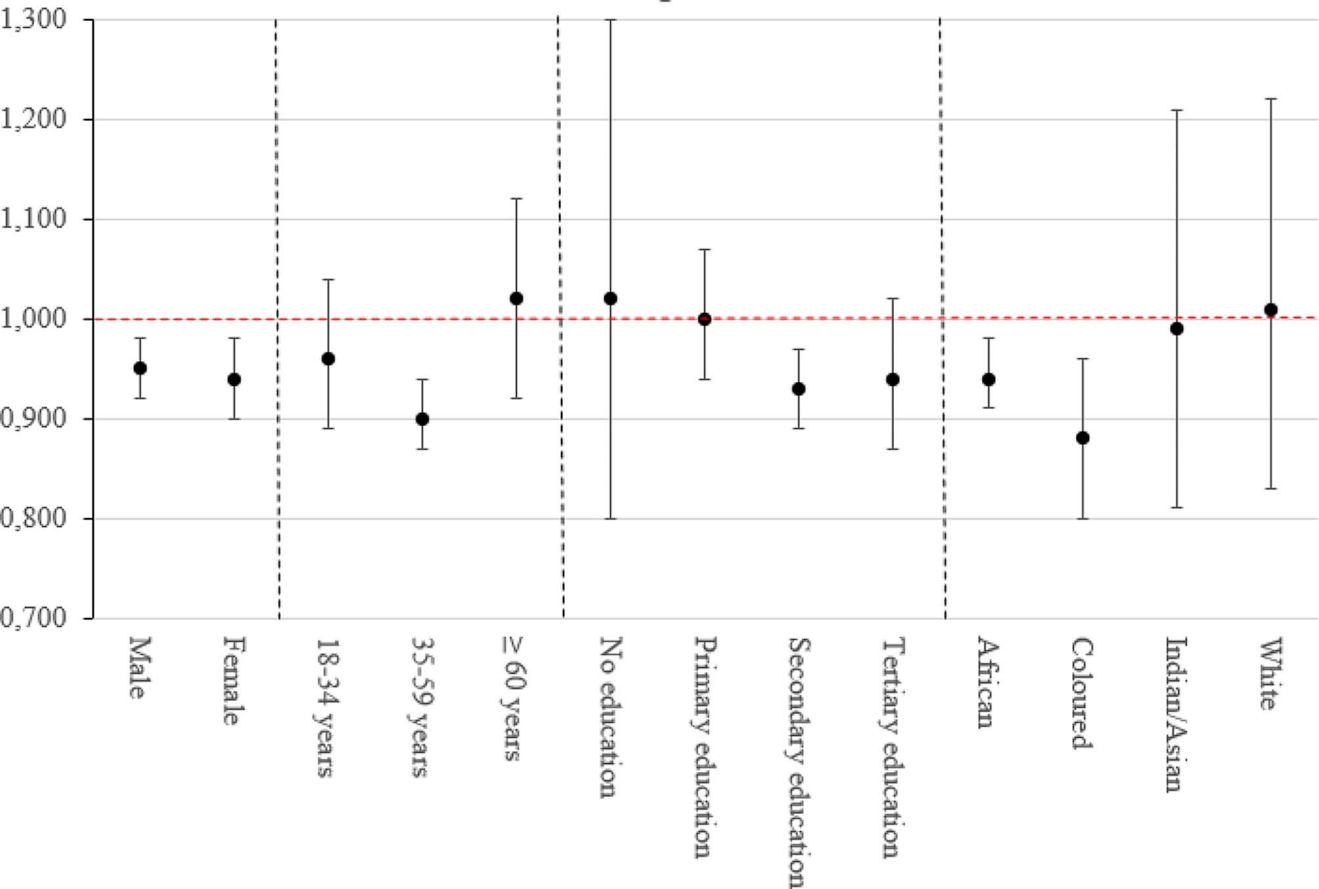
Adjusted associations (PR and 95% CI) between proximity to public green spaces and depressive symptoms stratified by sex, age groups, educational attainment, and population groups for those who reported a walking time of 15 min [reference group: >15 min]

With regards to the stratified analyses, Fig. 2 shows the association between proximity to public green spaces and depressive symptoms stratified by sex, age categories, educational attainment, and population group for those who reported a walking time of 15 min to the nearest public green space. The exact values of the PRs and 95% CIs are presented in Supplementary Table S2. By sex, slightly stronger inverse associations were observed for females (PR = 0.94; 95%CI = 0.90–0.98) than for males. Regarding age, we observed the strongest inverse association for those aged 35–59 years (PR = 0.90; 95%CI = 0.87–0.94) as compared to the other age categories. For population group, the strongest inverse association was observed for Coloured individuals (PR = 0.88; 95%CI = 0.80–0.96) as compared to other groups. By educational attainment, we observed stronger inverse associations for those who attained higher levels of education as compared to their counterparts.

Finally, the results of our sensitivity analyses are reported in Table S3 of the supplementary material. After increasing the PHQ-2 threshold score value to ≥ 3, our effect estimates were not statistically significant. Nonetheless, in all the sensitivity analyses, the PRs were overall similar to the ones illustrated in Fig. 1 and those reported in Table S1 (i.e., adjusted PRs ranged from 0.92 to 0.99).

## Discussion

In this study, we assessed the association between proximity to public green spaces and depressive symptoms among Gauteng Province residents, South Africa. Our findings indicate that public green spaces that were close to residential homes(within a 15 min walking distance) were associated with a prevalence reduction in depressive symptoms. In addition, we observed that this protective effect differed by subgroups of sex, age groups, educational attainment, and population group.

As highlighted by previous studies conducted in China [25] and the USA [26], access to green spaces is an associated factor for the utilisation of green spaces for active lifestyle behaviors such as physical activity, which ultimately improve mental health. Though there is limited evidence on the relationship between proximity to green spaces and depression, our finding of an inverse association between proximity to public green spaces and depressive symptoms is in line with a previous similar study in Spain which showed that access to outdoor spaces and nature views was associated with more positive emotions [39]. Furthermore, our finding is consistent with a similar study conducted in South Africa that used NDVI as a quantitative measure of green spaces [22].

When stratifying by sex, we observed slightly stronger inverse associations for proximity to green spaces and depressive symptoms for females than males. This is consistent with similar cross-sectional studies conducted in Lithuania [41]. Some previous studies have hypothesized that specific population groups such as females spend more time in the vicinity of their residential surroundings than their counterparts thus may explain this stronger beneficial protective effect [42]. In the global south context, this may be closely related to the gender roles attached to females and males. Females are usually more often involved in caregiving tasks such as domestic work and caring for children than males [43] thus may spend more time in their residential surroundings and in turn may potentially increase the utilisation of public green spaces. However, other studies reported stronger associations for males than females [44]. This inconsistency in findings further strengthens that green space and depression relationships among specific sub-populations may vary according to geographical, societal, or environmental contexts.

With regards to age, the protective effect of public green spaces against depressive symptoms was strongeramong middle-aged individuals from 35 to 59 years as compared to other age categories. A similar study conducted in Australia is in line with our finding [45], which observed an inverse association between green spaces and psychological distress among middle-aged adults. A probable explanation for this may be individuals of the aforementioned age group may be more health-conscious and are willing to engage in health-promoting behaviors such as physical activity as compared to the other age groups.

We observed stronger inverse associations for proximity to public green spaces and depressive symptoms among individuals with higher educational attainment (secondary education). A similar study conducted in South Africa corroborates this finding [22]. This may be because those with higher educational attainment tend to have higher incomes and reside in environments with easy access to public green spaces. In a national-scale analysis of urban green infrastructure conducted by Venter et al. (2020) in South Africa, they observed that those who resided in high-income areas had a closer proximity to parks as compared to those who resided in low-income areas [46]. Interestingly, it should be noted that we observed effect estimates (adjusted prevalence ratios) greater than one for individuals of lower educational attainment. A probable explanation of this finding may be associated with utilisation of the green spaces. Individuals with low educational attainment are more likely to reside in neighborhoods that are unsafe as compared to highly educated individuals. Such factors (including crime) may, in turn, affect their utilisation of public green spaces. A previous study conducted in South Africa showed aspects such as crime (safety) hindered the utilisation of available green spaces [47]. However, based on our study findings, it cannot be concluded that access to public green spaces may increase the risk of depressive symptoms among individuals with a lower educational attainment owing to the wide confidence intervals. As a result, there is a need for future research to assess other paramount aspects of public green spaces such as safety and their impact on depression occurrence.

In terms of population group, our findings from the stratified analysis indicate stronger protective effects of public green spaces against depressive symptoms for Coloured and Africans as compared to Indians/Asians and the White population group. Within the South African context, this may be a counter-intuitive finding because previous evidence has suggested that White population neighborhoods have closer proximity to public green spaces (a 700-meter average distance away from a public green space) as compared to the African population neighborhoods who live on average 2.6 km away from a public green space [46]. As a result, we would potentially expect stronger protective effects of public green spaces against depressive symptoms among White individuals as compared to Africans. A probable explanation for the observed stronger protective effects among African individuals than their White counterparts may be associated with factors related to the utilization of public green spaces (such as frequency of visits, distance traveled, and quality), which, unfortunately, we did not have information on in this study. Previous studies have demonstrated individuals may utilise public green spaces far away from their residential homes [48, 49]. Consequently, further research is warranted to understand the factors associated with the utilization of public green spaces among population groups in South Africa. Nevertheless, the observed stronger protective effect of public green spaces against depressive symptoms among the African population, reaffirms the need to provide public green spaces close to residential homes. Historically in South Africa, the African population group is viewed as a vulnerable group as compared to other population groups. Venter and colleagues observed a decrease in vegetation greenness from 1990 to 2018 in South African neighborhoods where Africans resided as compared to the neighborhoods of the other population groups where they observed an increase in vegetation greenness in the same time frame [46].

Our study has some limitations that should be taken into consideration when interpreting our results. Firstly, we relied on self-reported measures of proximity to public green spaces thus there is a potential for bias in our effect estimates as we did not have objective measures of proximity to public green spaces. However, self-reported measures of proximity to public green spaces are an example of a subjective indicator of green space quality. Such subjective green space quality indicators have been suggested by previous studies [44, 50] to potentially affect the utilisation of available public green spaces and may in turn influence the occurrence of depressive symptoms. Secondly, our assessment of proximity to public green spaces is limited to the residential homes and does not account for mobility to other green spaces beyond the residential homes. The limitations associated with using a static form of assessment like a residential home rather than a dynamic form that accounts for mobility are discussed elsewhere [51]. Furthermore, our assessment of public green spaces at the residential level does not include other measures of green spaces such as green spaces along streets which have been suggested by previous studies to play an important role in the association between green spaces and mental-health-related outcomes [52, 53]. Thirdly, there is a potential for residential confounding as we were unable to account for neighborhood socio-economic position and lifestyle behaviors (alcohol consumption and drug use)and neighborhood. However, previous similar studies that adjusted for these lifestyle behaviors did not observe a statistical significant attenuation in the effect estimates [54, 55]. In addition, we adjusted for socio-demographics which are potentially strongly correlated these lifestyle behaviors [56]. Lastly, our results need to be interpreted with caution because of the cross-sectional design of our study. Consequently, there is a need for longitudinal-based studies to further understand the relationship between green spaces and depressive symptoms.

Despite these limitations, our study has important strengths. Firstly, our study included an under-studied population (individuals from an African middle-income country), as a result our study provides paramount evidence as the majority of the previous evidence has originated from high-income countries. Secondly, we included a relatively large sample size thus having sufficient power to conduct several stratification analyses in subgroups of our study population.

## Conclusion

Our study provides some initial evidence that public green spaces close to residential homes may be associated with a reduction in the occurrence of depressive symptoms among urban populations in resource-constrained settings like South Africa. Our study findings support the need for continued efforts by stakeholders such as policy makers and urban planners to ensure easy accessibility to public green spaces as it may improve the mental well-being of urban residents.

### Electronic supplementary material

Below is the link to the electronic supplementary material.

Supplementary Material 1

## Data Availability

The dataset used and analysed during the current study are available from the corresponding author upon reasonable request.

## Abbreviations

CI: Confidence Intervals
GCRO: Gauteng City Region Observatory
NDVI: Normalised Difference Vegetation Index
PHQ: Patient Health Questionnaire
PR: Prevalence Ratios
WHO: World Health Organisation

## References

[1] WHO. World mental health report: transforming mental health for all. World Health Organization: Geneva; 2022.

[2] Craig A et al. The prevalence of probable depression and probable anxiety, and associations with adverse childhood experiences and socio-demographics: a national survey in South Africa. Front Public Health, 2022. 10. 10.3389/fpubh.2022.986531 PMC9650309 36388391

[3] Cassano P, Fava M (2002). Depression and public health: an overview. J Psychosom Res.

[4] Köhler CA (2018). Mapping risk factors for depression across the lifespan: an umbrella review of evidence from meta-analyses and mendelian randomization studies. J Psychiatr Res.

[5] Maier A (2021). Risk factors and protective factors of depression in older people 65+. A systematic review. PLoS ONE.

[6] Yang H et al. Association between Natural/Built Campus Environment and Depression among Chinese undergraduates: Multiscale evidence for the moderating role of socioeconomic factors after Controlling for Residential Self-Selection. Front Public Health, 2022. 10. 10.3389/fpubh.2022.844541 PMC9037627 35480591

[7] Sui Y, Ettema D, Helbich M (2022). Longitudinal associations between the neighborhood social, natural, and built environment and mental health: a systematic review with meta-analyses. Health Place.

[8] Geneshka M (2021). Relationship between Green and Blue Spaces with Mental and Physical Health: a systematic review of Longitudinal Observational studies. Int J Environ Res Public Health.

[9] Jennings V, Bamkole O (2019). The relationship between Social Cohesion and Urban Green Space: An Avenue for Health Promotion. Int J Environ Res Public Health.

[10] Knobel P (2021). Quality of urban green spaces influences residents’ use of these spaces, physical activity, and overweight/obesity. Environ Pollut.

[11] Shen J (2021). Green Space and Physical Activity in China: a systematic review. Sustainability.

[12] Liu L (2022). Restorative benefits of urban green space: physiological, psychological restoration and eye movement analysis. J Environ Manage.

[13] Høj SB (2021). Relative ‘greenness’ and not availability of public open space buffers stressful life events and longitudinal trajectories of psychological distress. Health Place.

[14] Diener A, Mudu P (2021). How can vegetation protect us from air pollution? A critical review on green spaces’ mitigation abilities for air-borne particles from a public health perspective - with implications for urban planning. Sci Total Environ.

[15] Junior DPM, Bueno C, Silva CMda (2022). The Effect of Urban Green spaces on reduction of Particulate Matter Concentration. Bull Environ Contam Toxicol.

[16] Dzhambov A, Dimitrova D (2014). Urban green spaces’ effectiveness as a psychological buffer for the negative health impact of noise pollution: a systematic review. Noise Health.

[17] Schäffer B (2020). Residential green is associated with reduced annoyance to road traffic and railway noise but increased annoyance to aircraft noise exposure. Environ Int.

[18] Borroni E (2022). Air pollution exposure and depression: a comprehensive updated systematic review and meta-analysis. Environ Pollut.

[19] Hegewald J et al. Traffic noise and Mental Health: a systematic review and Meta-analysis. Int J Environ Res Public Health, 2020. 17(17). 10.3390/ijerph17176175 PMC7503511 32854453

[20] Roberts H (2019). The effect of short-term exposure to the natural environment on depressive mood: a systematic review and meta-analysis. Environ Res.

[21] Markevych I (2017). Exploring pathways linking greenspace to health: theoretical and methodological guidance. Environ Res.

[22] Tomita A (2017). Green environment and incident depression in South Africa: a geospatial analysis and mental health implications in a resource-limited setting. Lancet Planet Health.

[23] Zewdie HY (2022). The association between urban greenspace and psychological health among young adults in Addis Ababa, Ethiopia. Environ Res.

[24] WHO. Green and blue spaces and mental health: new evidence and perspectives for action. WHO Regional Office for Europe: Copenhagen; 2021.

[25] Zhang W, et al. Factors affecting the use of urban green spaces for physical activities: views of young urban residents in Beijing. Volume 14. Urban Forestry & Urban Greening; 2015. pp. 851–7. 4.

[26] Cohen DA (2007). Contribution of Public Parks to Physical Activity. Am J Public Health.

[27] UN-Habitat. South Africa. 2023 [cited 2023 03/04/2023]; Available from: https://unhabitat.org/south-africa.

[28] Girma Y et al. *Urban green spaces supply in rapidly urbanizing countries: The case of Sebeta Town, Ethiopia* Remote Sensing Applications: Society and Environment, 2019. 13: p. 138–149.

[29] Witwatersrand Uot. *Gauteng City-Region Observatory*. 2023 [cited 2023 8 May ]; Available from: https://wiredspace.wits.ac.za/communities/20bd3a37-20c9-435e-af32-f36f28cdd662.

[30] GCRO. Quality of Life V (2017/18) survey viewer. 2021 [cited 2023 8 May 2023]; Available from: https://www.gcro.ac.za/data-gallery/quality-of-life/detail/quality-life-v-201718-survey-viewer/.

[31] De Kadt J (2021). Quality of Life survey 6 (2020/21): overview report.

[32] GCRO. QoL V (2017/18) survey: preliminary results results launch. Gauteng City-Region Observatory; 2019.

[33] Levis B (2020). Accuracy of the PHQ-2 alone and in combination with the PHQ-9 for screening to detect major depression: systematic review and meta-analysis. JAMA.

[34] Gascon M (2015). Mental health benefits of long-term exposure to residential green and blue spaces: a systematic review. Int J Environ Res Public Health.

[35] sa S. *SA population reaches 58,8 million* 2019 [cited 2023 11/08/2023]; Available from: https://www.statssa.gov.za/?p=12362.

[36] Kon ZR, Lackan N (2008). Ethnic disparities in access to care in post-apartheid South Africa. Am J Public Health.

[37] Bank W. *Republic of South Africa Systematic Country Diagnostic: An Incomplete Transition Overcoming the Legacy of Exclusion in South Africa*. 2018.

[38] Kroenke K, Spitzer RL, Williams JB (2003). The Patient Health Questionnaire-2: validity of a two-item depression screener. Med Care.

[39] Pouso S (2021). Contact with blue-green spaces during the COVID-19 pandemic lockdown beneficial for mental health. Sci Total Environ.

[40] Sarkar C (2021). Characteristics of the residential environment and their Association with Depression in Hong Kong. JAMA Netw Open.

[41] Reklaitiene R (2014). The relationship of green space, depressive symptoms and perceived general health in urban population. Scand J Public Health.

[42] Maas J (2006). Green space, urbanity, and health: how strong is the relation?. J Epidemiol Community Health.

[43] van der Gaag N et al. *State of the World’s Fathers: Centering care in a world in crisis*, D. Washington, Editor. 2023, Equimundo.

[44] Richardson EA, Mitchell R (2010). Gender differences in relationships between urban green space and health in the United Kingdom. Soc Sci Med.

[45] Astell-Burt T, Feng X, Kolt GS (2013). Mental health benefits of neighbourhood green space are stronger among physically active adults in middle-to-older age: evidence from 260,061 australians. Prev Med.

[46] Venter ZS, et al. Green apartheid: urban green infrastructure remains unequally distributed across income and race geographies in South Africa. Volume 203. Landscape and Urban Planning; 2020. p. 103889.

[47] Sabirah A et al. Considering the natural environment in the creation of child-friendly cities: implications for children’s Subjective Well-Being. Child Indic Res, 2019. 12(2).

[48] Yang Y, Diez-Roux AV (2012). Walking Distance by trip purpose and Population subgroups. Am J Prev Med.

[49] Rigolon A (2021). Green Space and Health Equity: a systematic review on the potential of Green Space to Reduce Health disparities. Int J Environ Res Public Health.

[50] Nguyen PY et al. Green Space Quality and Health: a systematic review. Int J Environ Res Public Health, 2021. 18(21). 10.3390/ijerph182111028 PMC8582763 34769549

[51] Zhang L (2021). Assessing individual environmental exposure derived from the spatiotemporal behavior context and its impacts on mental health. Health Place.

[52] Helbich M (2019). Using deep learning to examine street view green and blue spaces and their associations with geriatric depression in Beijing, China. Environ Int.

[53] Wang R (2019). Urban greenery and mental wellbeing in adults: cross-sectional mediation analyses on multiple pathways across different greenery measures. Environ Res.

[54] Klompmaker JO (2019). Associations of combined exposures to surrounding green, air pollution and traffic noise on mental health. Environ Int.

[55] Abraham Cottagiri S (2022). Increased urban greenness associated with improved mental health among middle-aged and older adults of the Canadian longitudinal study on aging (CLSA). Environ Res.

[56] Kabisch N. *The Influence of Socio-economic and Socio-demographic Factors in the Association Between Urban Green Space and Health*, in *Biodiversity and Health in the Face of Climate Change*, M.R. Marselle, Editors. 2019, Springer International Publishing: Cham. p. 91–119.

