# Supplementary material for: Proximity to public green spaces and depressive symptoms among South African residents: a population-based study
Source: BMC Public Health. 2024 Mar 29;24:925. doi: 10.1186/s12889-024-18385-1 (PMC10981334; doi:10.1186/s12889-024-18385-1)

Supplementary material

***Proximity to public green spaces and depressive symptoms among South African residents: A population-based study***

*Authors: Busisiwe Shezi*, Hilbert Mendoza*, Darshini Govindasamy, Lidia Casas, Yusentha Balakrishna, Jason Bantjes, Renée Street*

**Shared co-first authorship*

**Table of contents**

[**TABLES** 2](#_Toc159450372)

[Table S 1: Prevalence Ratios (PRs) and their 95% confidence intervals (CI) with increasing covariate adjustment for the association between proximity to public green spaces and depressive symptoms 2](#_Toc159450373)

[Table S 2:Adjusted associations (PR and 95% CI) between proximity to public green spaces and depressive symptoms, stratified by sex, age, and educational attainment 2](#_Toc159450374)

[≥60 years (n=3,186) 2](#_Toc159450375)

[Table S 3: Prevalence Ratios (PRs) and their 95% confidence intervals (CI) for the association between proximity to public green spaces and depressive symptoms when a PHQ-2 score threshold value of 3 is applied, depression score as a continuous variable, seasonal variations adjusted for as a potential confounder, age in its continuous form, and the full study population including those with missing covariate data. 3](#_Toc159450376)

[**FIGURES** 4](#_Toc159450377)

[Figure S 1: Directed acyclic graph of potential confounders and mediators in the association between proximity to public green spaces and depressive symptoms 4](#_Toc159450378)

[Figure S 2: A map of the nine municipalities of Gauteng Province and its relative location in Africa 5](#_Toc159450379)

**TABLES**

Table S 1: Prevalence Ratios (PRs) and their 95% confidence intervals (CI) with increasing covariate adjustment for the association between proximity to public green spaces and depressive symptoms

| **Green space metric** | **M1** | **M2** | **M3** |
| --- | --- | --- | --- |
|  | **PR (95% CI)** | **PR (95% CI)** | **PR (95% CI)** |
| **Proximity to public green spaces**  >15 minutes (Ref)  Within 15 minutes | 0.90 (0.88-0.92) | 0.93 (0.91-0.95) | 0.94 (0.92-0.96) |

M1: Accounted for between-area variability by including a random term of municipality

M2: Adjusted for M1, sex, age, and population group

M3: Adjusted for M2 and educational attainment

Table S 2:Adjusted associations (PR and 95% CI) between proximity to public green spaces and depressive symptoms, stratified by sex, age, and educational attainment

|  | **Proximity to public green spaces [reference group: > 15 minutes]** |
| --- | --- |
|  | **PR^1^ (95% CI)** |
| **Sex**  Female (n=12,991)  Male (n=11,350) | 0.94 (0.90-0.98)  0.95 (0.92-0.98) |
| **Age categories**  18-34 years (n=9,752)  35-59 years (n=11,403)  ≥60 years (n=3,186) | 0.96 (0.89-1.04)  0.90 (0.87-0.94)  1.02 (0.92-1.12) |
| **Educational attainment**  No education (n=683)  Primary education (n=2,372)  Secondary education (n=14,828)  Tertiary education (n=6,458) | 1.02 (0.80-1.27)  1.01 (0.95-1.07)  0.93 (0.89-0.97)  0.94 (0.87-1.03) |
| **Population group**  African (n=20,618)  Coloured (n=866)  Indian/Asian (n=347)  White (n=2,510) | 0.94 (0.91-0.98)  0.88 (0.80-0.96)  0.99 (0.81-1.21)  1.01 (0.83-1.22) |

^1^Accounted for the municipality as a random effect, adjusted for sex, age, population group, and educational attainment.

Table S 3: Prevalence Ratios (PRs) and their 95% confidence intervals (CI) for the association between proximity to public green spaces and depressive symptoms when a PHQ-2 score threshold value of 3 is applied, depression score as a continuous variable, seasonal variations adjusted for as a potential confounder, age in its continuous form, and the full study population including those with missing covariate data.

| **Sensitivity analysis** | **Green space metric** | **PR^1^ (95% CI)** |
| --- | --- | --- |
| Depression score with 3 as optimal cut-off (n=24,341) | **Proximity to public green spaces**  >15 minutes (Ref)  Within 15 minutes | 0.98 (0.96-1.00) |
| Depression score as a continuous variable (n=24,341) | **Proximity to public green spaces**  >15 minutes (Ref)  Within 15 minutes | 0.93 (0.90-0.97) |
| Seasonal differences as an adjusted potential confounder (n=24,341) | **Proximity to public green spaces**  >15 minutes (Ref)  Within 15 minutes | 0.96 (0.93-0.98) |
| Age adjusted for in the main model as a continuous variable (n=24,341) | **Proximity to public green spaces**  >15 minutes (Ref)  Within 15 minutes | 0.94 (0.92-0.96) |
| Full population including those with missing covariate data (n=24,804) | **Proximity to public green spaces**  >15 minutes (Ref)  Within 15 minutes | 0.96 (0.91-0.99) |

^1^accounted for between area-variability by including the municipality as a random effect, adjusted for sex, age, population group, and educational attainment.

# **FIGURES**

Figure S 1: A map of the nine municipalities of Gauteng Province and its relative location in Africa


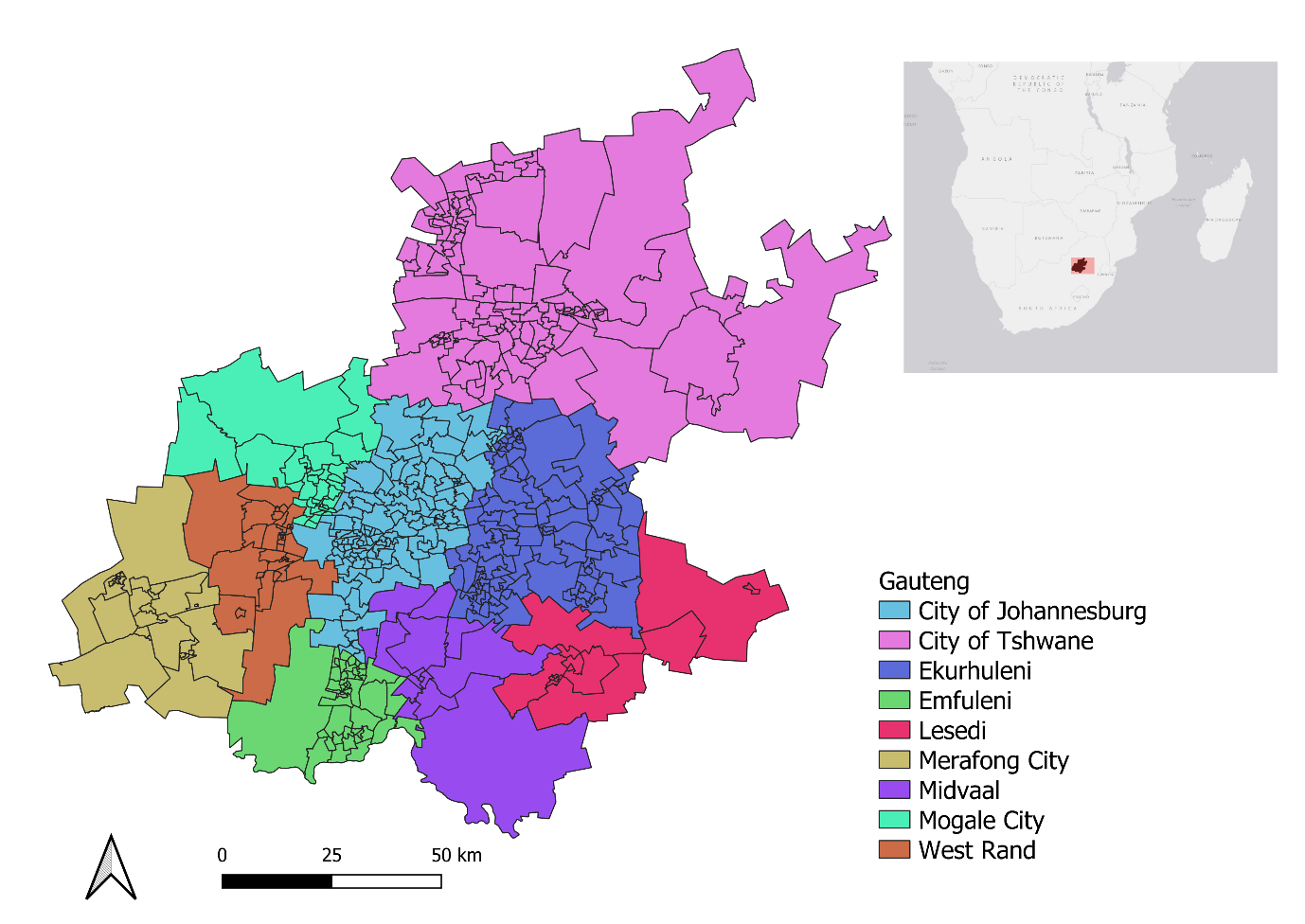


Figure S 2: Directed acyclic graph of potential confounders and mediators in the association between proximity to public green spaces and depressive symptoms


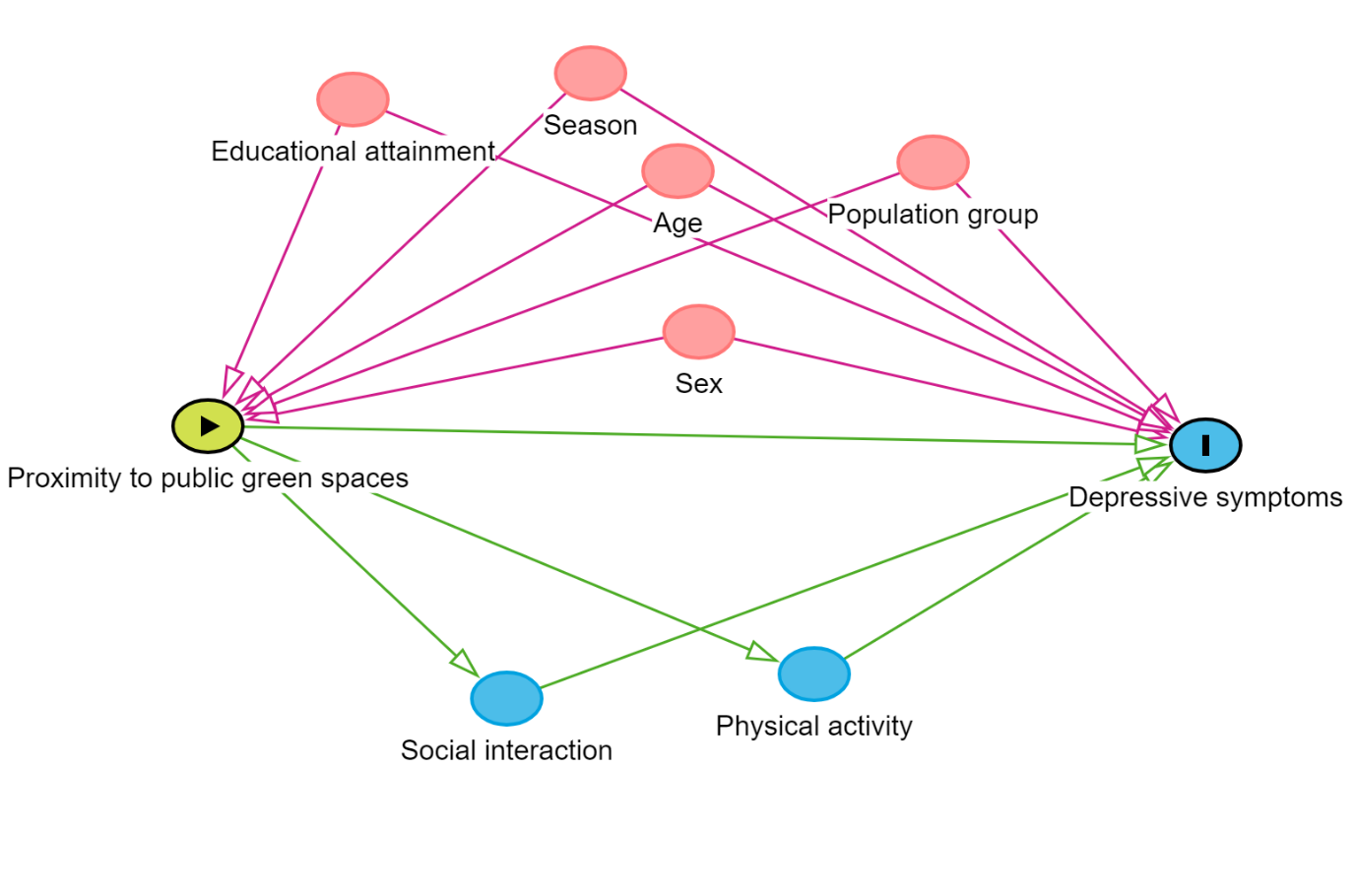

Supplement: Supplementary file 1 — Supplementary Material 1 [file 12889_2024_18385_MOESM1_ESM.docx]
